# Rapunzel Syndrome in a Child With Sensory Feeding Difficulties and Attention-Deficit Hyperactivity Disorder

**DOI:** 10.7759/cureus.103282

**Published:** 2026-02-09

**Authors:** Richard M Lurshay, Andrew Dunbar, Ousama Suliman, Nicholas Lipscomb

**Affiliations:** 1 Paediatrics and Child Health, South West Acute Hospital, Enniskillen, GBR

**Keywords:** magnetic resonance enterography, pediatric surgery, pediatric weight loss, rapunzel syndrome, sensory feeding difficulties, trichobezoar, trichophagia

## Abstract

Rapunzel syndrome is a rare form of trichobezoar characterized by the extension of a gastric hair mass beyond the pylorus into the small intestine. It is most commonly reported in adolescents with underlying psychiatric illness. We describe a diagnostically challenging case in a younger child with neurodevelopmental vulnerability and sensory feeding difficulties.

A 10-year-old girl with attention-deficit hyperactivity disorder, sensory aversion to food textures, and heterozygous chromosomal deletions presented with progressive weight loss, anxiety around eating, and intermittent abdominal pain. Initial investigations for faltering growth and anemia were inconclusive. Magnetic resonance enterography, performed to exclude inflammatory bowel disease, demonstrated a large intragastric filling defect extending into the duodenum. Surgical exploration confirmed a large trichobezoar consistent with Rapunzel syndrome, which was successfully removed via laparotomy.

This case highlights how Rapunzel syndrome may masquerade as a functional or sensory feeding disorder and emphasizes the value of magnetic resonance enterography as a radiation-free diagnostic modality in pediatric patients with unexplained weight loss.

## Introduction

Trichobezoars are rare gastrointestinal masses composed predominantly of ingested hair and are typically associated with trichotillomania and trichophagia. Rapunzel syndrome represents an uncommon and more severe manifestation, defined by extension of the trichobezoar beyond the stomach into the small intestine. Pediatric cases are infrequently reported and often present with non-specific symptoms, such as abdominal pain, anemia, and weight loss, which can delay diagnosis. If unrecognized, trichobezoars and Rapunzel syndrome can rarely result in serious complications, including partial or complete intestinal obstruction, gastrointestinal perforation, and peritonitis [1,2].

Children with neurodevelopmental conditions or sensory feeding difficulties present a particular diagnostic challenge, as gastrointestinal symptoms may be attributed to behavioral or functional etiologies. Imaging plays a central role in diagnosis; however, most reported cases rely on ultrasound or computed tomography. The role of magnetic resonance enterography in the diagnosis of trichobezoar remains underreported. This case illustrates an atypical presentation of Rapunzel syndrome and emphasizes the importance of maintaining diagnostic vigilance in children with complex feeding behaviors.

## Case presentation

A 10-year-old girl presented with an 8-month history of progressive weight loss, intermittent, sharp left upper quadrant abdominal pain, and increasing anxiety surrounding eating. Her medical history included attention-deficit hyperactivity disorder, with stimulant medication withheld due to weight loss, sensory aversion to food textures, and heterozygous chromosomal deletions involving 17p12 and 9q33.3.

Despite expressing hunger, she frequently refused meals, particularly solid foods, and relied predominantly on liquids. Over eight months, her weight declined from the 60th centile to the 1st centile on the UK-WHO growth chart. Physical examination revealed a firm, irregular, intermittently palpable upper abdominal mass.

**Figure 1:**
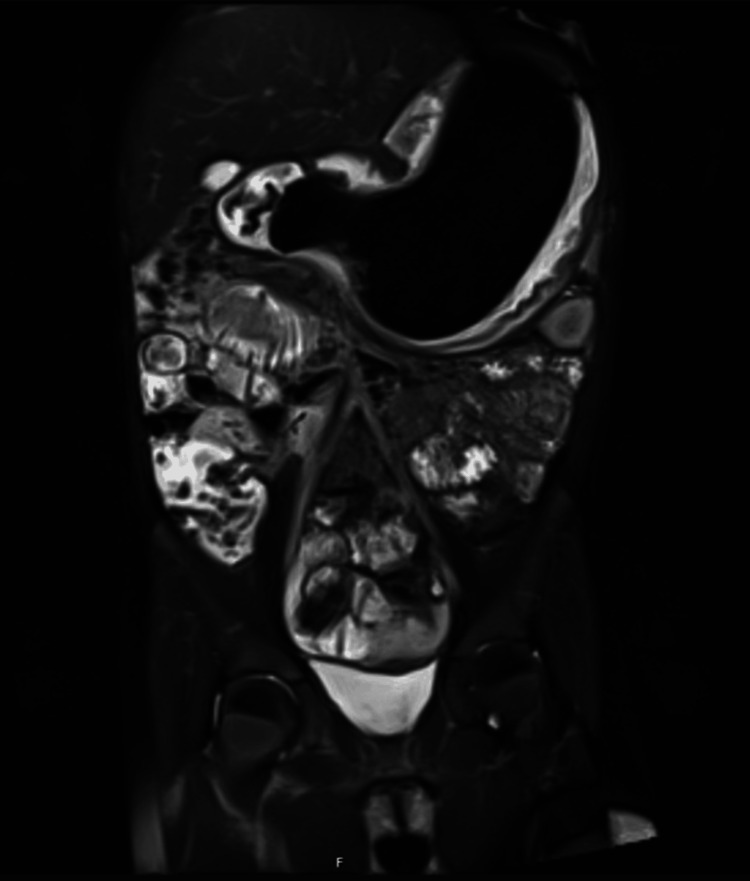
Magnetic resonance enterography (coronal T2-weighted image) demonstrating a large, well-defined intragastric filling defect that appears markedly hypointense The mass measures approximately 15–16 cm in length and extends into the proximal duodenum.

Laboratory investigations demonstrated iron deficiency anemia with a hemoglobin concentration of 113 g/L. Inflammatory markers and nutritional screening were within normal limits. Differential diagnoses included sensory feeding disorder, eating disorder, inflammatory bowel disease, and foreign body ingestion. An abdominal ultrasound examination was performed, but was technically limited due to excessive overlying bowel gas, and no significant abnormalities were identified. Given ongoing clinical concern and the need to evaluate for inflammatory bowel disease, magnetic resonance enterography was subsequently performed. Magnetic resonance enterography demonstrated a 15 to 16 cm intragastric filling defect extending into the duodenum, consistent with a trichobezoar (Figure 1).

**Figure 2:**
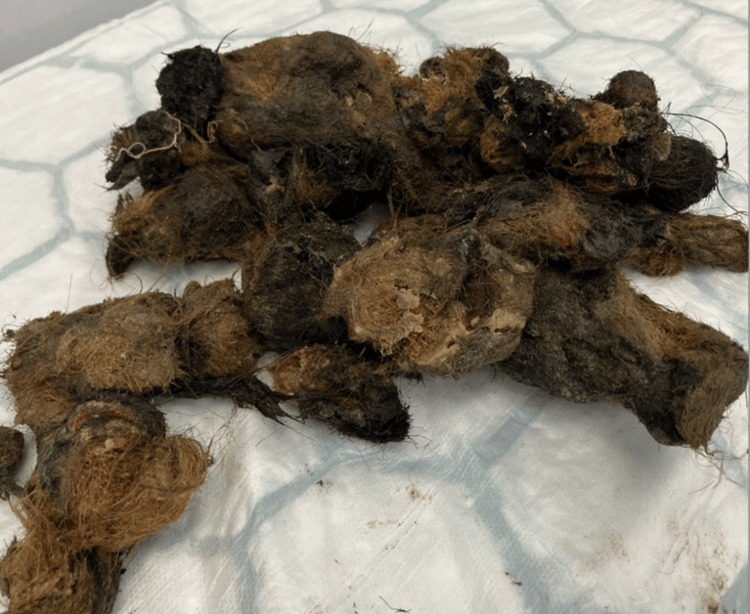
Intraoperative photograph showing the extracted trichobezoar in its entirety

The patient underwent a planned laparotomy on July 26, 2025. A laparoscopic approach was initially undertaken, with a 5-millimeter port inserted using an open technique. Laparoscopic assessment confirmed a large trichobezoar occupying the stomach with extension through the pylorus into the duodenum, consistent with Rapunzel syndrome. Given the size and extent of the bezoar, a decision was made to proceed with a left upper transverse incision, allowing complete and safe removal of the trichobezoar. The mass, composed of hair and vegetable fibers, was extracted in its entirety without complication, and the gastrotomy was closed (Figure 2).

Postoperatively, the patient recovered uneventfully. Oral feeding was gradually reintroduced under dietetic supervision. Behavioral therapy was initiated to address trichotillomania and trichophagia, with ongoing follow-up through Child and Adolescent Mental Health Services. At three-month follow-up, her weight had improved to the 24th centile on the UK-WHO growth chart, with resolution of abdominal symptoms and no evidence of recurrence.

## Discussion

This case demonstrates an atypical presentation of Rapunzel syndrome in a child without overt psychiatric illness, where sensory feeding difficulties and neurodevelopmental factors obscured the underlying diagnosis. The clinical features closely resembled a functional feeding disorder or early eating disorder, contributing to diagnostic delay. Although alopecia or bald patches are frequently reported in patients with trichotillomania, no visible hair loss was noted in this patient, further contributing to diagnostic delay and highlighting that Rapunzel syndrome may occur in the absence of classical external signs.

Physical examination findings were intermittent, and laboratory investigations were largely non-specific. In this case, magnetic resonance enterography was not selected as a first-line investigation for suspected trichobezoar. An initial abdominal ultrasound was performed, but it was technically limited and non-diagnostic. Magnetic resonance enterography was subsequently undertaken for an alternative clinical indication and provided clear delineation of the intragastric mass without exposure to ionizing radiation. While ultrasound, computed tomography, and endoscopy remain commonly reported diagnostic modalities for bezoars, this case highlights the complementary role of magnetic resonance enterography when initial imaging is inconclusive, particularly in pediatric patients. Magnetic resonance enterography proved decisive in identifying the intragastric pathology. While computed tomography is more commonly reported in the diagnosis of bezoars, magnetic resonance enterography offers excellent soft tissue characterization without ionizing radiation, making it particularly suitable for pediatric patients [3].

This case is notable not for the rarity of Rapunzel syndrome alone, but for its diagnostic pathway. The patient’s sensory feeding difficulties and neurodevelopmental profile led to symptoms being initially attributed to behavioral causes, delaying recognition of an underlying surgical condition. Additionally, the use of magnetic resonance enterography as a diagnostic modality highlights its value in evaluating unexplained pediatric weight loss when initial investigations are inconclusive.

Although the patient’s chromosomal deletions are not directly associated with bezoar formation, associated sensory and behavioral factors may have contributed indirectly to hair ingestion and delayed symptom recognition. This case underscores the importance of maintaining a broad differential diagnosis and a multidisciplinary approach in children with complex feeding presentations.

## Conclusions

Rapunzel syndrome should be considered in pediatric patients presenting with unexplained weight loss and abdominal symptoms, even in the absence of classic psychiatric features. This case illustrates how children with neurodevelopmental and sensory feeding difficulties may present with atypical features, increasing the risk of diagnostic delay.

While conclusions drawn from a single case are inherently limited, this report highlights the potential diagnostic value of magnetic resonance enterography when initial investigations are inconclusive and underscores the importance of a multidisciplinary approach to management.
